# Data on the fluoride adsorption from aqueous solutions by metal-organic frameworks (ZIF-8 and Uio-66)

**DOI:** 10.1016/j.dib.2018.08.159

**Published:** 2018-08-31

**Authors:** Bahram Kamarehie, Zahra Noraee, Ali Jafari, Mansour Ghaderpoori, Mohammad Amin Karami, Afshin Ghaderpoury

**Affiliations:** a Department of Environmental Health Engineering, School of Health and Nutrition, Lorestan University of Medical Sciences, Khorramabad, Iran; b Nutritional Health Research Center, Lorestan University of Medical Sciences, Khorramabad, Iran; c Student Research Committee, Shahid Beheshti University of Medical Sciences, Tehran, Iran

**Keywords:** Fluoride, Aqueous solution, Adsorption, Metal-organic framework

## Abstract

The variables examined were initial fluoride concentration, ZIF-8 and Uio-66 dosage, pH, and contact time. The residual concentration of fluoride was measured by a spectrophotometer. According to BET, the specific surface area of the ZIF-8 and Uio-66 was 1050 m^2^/g and 800 m^2^/g, respectively. Total pore volume and average pore diameter of the ZIF-8 and Uio-66 were 0.57 cm^3^/g, 0.45 cm^3^/g and 4.5 nm, 3.2 nm, respectively. The best pH for fluoride adsorption was neutral conditions. By increasing the ZIF-8 and Uio-66 dose, the fluoride uptake increased at first, but then decreased. Also, the maximum adsorption for ZIF-8 and Uio-66 was observed in adsorbent dose 0.2 and 0.6 g/L, respectively. The best model for describing kinetic and isotherms of fluoride adsorption were the pseudo-second-order model and Langmuir isotherm model, respectively. Based on the Langmuir model, the adsorption capacity of fluoride by ZIF-8 and Uio-66 was reported to be 25 mg/g and 20 mg/g, respectively.

**Specifications table**

**Table:** 

Subject area	*Water treatment*
More specific subject area	*Adsorption*
Type of data	*Figures and tables*
How data was acquired	*Spectrophotometer (UV-UVIS, 570 nm)*
Data format	*Analyzed*
Experimental factors	*The main variables examined were initial concentration of fluoride, ZIF-8 and Uio-66 dosage, pH, and contact time. At first, a stock solution of fluoride (NaF, 1000 mg/l) was made and stored under standard conditions. At the end of the experiments, the remaining adsorbents were separated using a centrifuge (3000 rpm, 5 min). After separation, the residual fluoride was measured by a spectrophotometer DR-5000.*
Experimental features	*ZIF-8 was first synthesized. In the second step, the absorbent of Uio-66 was synthesized. After synthesizing adsorbents, the general characterization of the adsorbent was determined based on XRD, SEM, and BET.*
Data source location	*Khorramabad, Lorestan University of Medical Sciences, Iran*
Data accessibility	*Data are included in this article*
Related research article	*A.A. Mohammadi, A. Alinejad, B. Kamarehie, S. Javan, A. Ghaderpoury, M. Ahmadpour, M. Ghaderpoori. Metal organic framework Uio-66 for adsorption of methylene blue dye from aqueous solutions. Int J Environ Sci Te. 14 (2017) 1959–1968.*

**Value of the data**

● The dataset will be useful for the application of the metal-organic framework in the fluoride adsorption from aqueous solutions.
● The data of this project can be used to improve drinking water quality by the authorities.
● Information from this data, including, kinetic and isotherm constants, will be informative for predicting and modelling the adsorption capacity and mechanism of fluoride uptake by ZIF-8 and Uio-66.
● The characterization data of the ZIF-8 and Uio-66 are useful for the scientific community to complete the studies for emerging absorbers.

## 1 Data

**Fig. 1:**
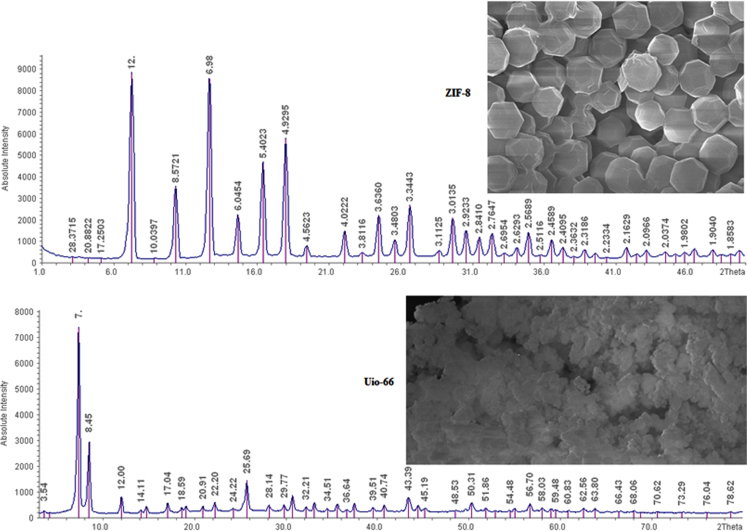
Results of XRD and SEM of synthesized ZIF-8 and Uio-66.

**Fig. 2:**
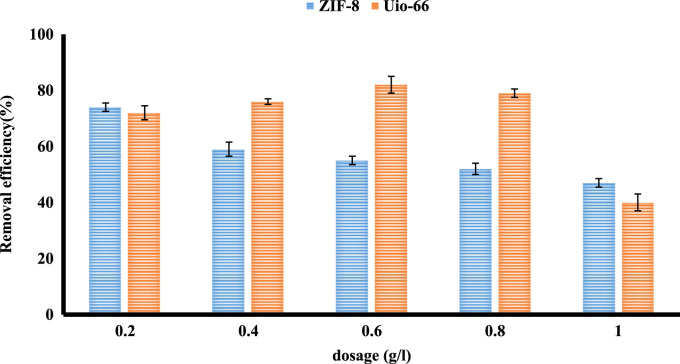
The effect of adsorbent dose of ZIF-8 and Uio-66 on fluoride adsorption.

**Fig. 3:**
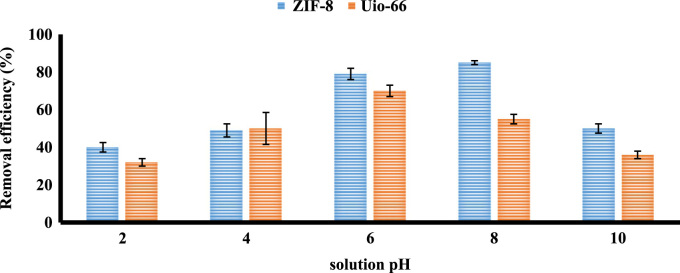
The effect of solution pH of ZIF-8 and Uio-66 on fluoride adsorption.

**Fig. 4:**
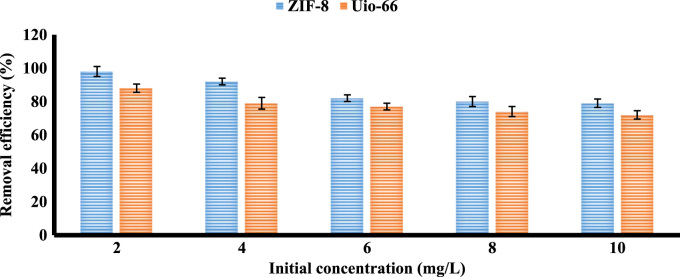
The effect of initial concentration of ZIF-8 and Uio-66 on fluoride adsorption.

The XRD and SEM results of synthesized ZIF-8 and Uio-66 are shown in Fig. 1. All adsorption experiments were performed in triplicate. Results of BET present in Table 1. The effects of an adsorbent dose of ZIF-8 and Uio-66 on fluoride adsorption are presented in Fig. 2. The effects of solution pH of ZIF-8 and Uio-66 on fluoride adsorption are depicted in Fig. 3. The effects of the initial concentration of ZIF-8 and Uio-66 on fluoride adsorption are shown in Fig. 4. Calculated parameters of kinetic models for the fluoride adsorption onto ZIF-8 and Uio-66 are summarized in Table 2. As illustrated in Table 2, the pseudo-second-order model for ZIF-8 and Uio-66 has the highest *R*^2^ (coefficient of determination). As a result, the model was the most suitable model to express the kinetics of the fluoride adsorption onto ZIF-8 and Uio-66. Calculated parameters of isotherm models for the fluoride adsorption onto ZIF-8 and Uio-66 are given in Table 3. As illustrated in Table 3, the Langmuir isotherm model for ZIF-8 and Uio-66 has the highest *R*^2^. As a result, the model was the most suitable model to express the isotherm of the fluoride adsorption onto ZIF-8 and Uio-66.

**Table 1:** Results of BET for synthesized ZIF-8 and Uio-66.

**Adsorbent**	**SA**_**BET**_**(m**^**2**^**g**^**−1**^**)**	**SA**_**Langmuir**_**(m**^**2**^** g**^**−1**^**)**	**Total pore volume (m**^**3**^**g**^**−1**^**)**	**Mean pore diameter (nm)**
**ZIF-8**	1050	1150	0.57	4.5
**Uio-66**	800	970	0.45	3.2

**Table 2:** Calculated parameters of kinetic models for the fluoride adsorption onto ZIF-8 and Uio-66.

**Kinetics (ZIF-8)**	**Constants**	**F concentration (**mg l^−1^**)**		**kinetics (Uio-66)**	**Constants**	**F concentration (**mg l^−1^**)**	
		**2**	**4**			**2**	**4**
**Pseudo-First-Order**	*k*_1_	0.0156	0.0167	**Pseudo-First-Order**	*k*_1_	0.0187	0.0196
	*R*^2^	0.745	0.667		*R*^2^	0.765	0.798
	*q*_cal_	5.678	12.674		*q*_cal_	6.576	14.243
**Pseudo-Second-Order**	*k*_2_	0.0443	0.0465	**Pseudo-Second-Order**	*k*_2_	0.0456	0.0645
	*R*^2^	0.895	0.834		*R*^2^	0.886	0.978
	*q_e_*_(cal)_	12.6	17.5		*q_e_*_(cal)_	13	15.7

**Table 3:** Calculated parameters of isotherm models for the fluoride adsorption onto ZIF-8 and Uio-66.

**Isotherm (ZIF-8)**	**Constants**	**F concentration (**mg l^−1^**)**		**Isotherm (Uio-66)**	**Constants**	**F concentration (**mg l^−1^**)**	
		**2**	**4**			**2**	**4**
**Freundlich**	*n*	3.5	5.3	**Freundlich**	*n*	2.7	3.6
	R^2^	0.775	0.845		*R*^2^	0.675	0.778
	*K_F_*	18.5	18.9		*K_F_*	19	19.8
**Langmuir**	*K_L_*	1.5	1.8	**Langmuir**	*K_L_*	0.576	0.657
	R^2^	0.897	0.898		*R*^2^	0.879	0.898
	*q_m_*	25	29		*q_m_*	20	28

## 2 Experimental design, materials, and methods

### 2.1 Materials

Chemicals used were zinc nitrate hexahydrate, methanol, N, N-dimethylformamide, zirconium chloride, 2-methylimidazole, and terephthalic acid. All the above-mentioned materials are prepared with high purity. The Materials were purchased from MERK and Sigma-Aldrich companies.

### 2.2 Synthesis of ZIF-8 and Uio-66

ZIF-8 was first synthesized. This adsorbent was synthesized based on the procedure presented by two previous works [1], [2]. In the second step, the absorbent of Uio-66 was synthesized. For the synthesis of this absorbent, previous studies were used [3], [4]. After synthesizing adsorbents, the general characterization of the adsorbent was determined based on XRD, SEM, and BET.

### 2.3 The adsorption experiments

Fluoride adsorption was investigated by the metal-organic frameworks of ZIF-8 and Uio-66. The experiments were performed in batch conditions. The main variables examined were initial concentration of fluoride, ZIF-8 and Uio-66 dosage, solution pH, and contact time. At first, a stock solution of fluoride (NaF, 1000 mg/l) was made and stored under standard conditions. An adsorbent of ZIF-8 and Uio-66 was added to 50 ml of fluoride solution. The solution pH was adjusted using NaOH [0.1 N] and H_2_SO_4_ [0.1 N]. At the end, the used adsorbents were separated using a centrifuge (3000 rpm, 5 min). After separation, the final concentration of fluoride was measured by a spectrophotometer DR-5000 (UV-UVIS, 570 nm) [5], [6], [7], [8]. Finally, fluoride adsorbed (*q_e_*, mg/g) and the removal efficiency (%) on the ZIF-8 and Uio-66 was computed based on Eqs. (1), (2), respectively [9], [10]:

(1) $$q_{e},mg/g=\frac{\left(C_{o}(mg/l)-C_{e}(mg/l)V(L\right)}{m(g)}$$

(2) $$R,\%=\frac{\left(C_{0}(mg/l)-C_{t}(mg/l)\right)}{C_{0}(mg/l)}$$

where, *C*_*o*_ *and C*_*e*_*, and C*_*t*_ are an initial, equilibrium, and final concentration, respectively. *V* and m are the volume of solution and the adsorbent weight, respectively [11], [12], [13], [14], [15], [16], [17], [18], [19], [20], [21], [22], [23].

## References

[1] Massoudinejad M., Ghaderpoori M., Shahsavani A., Jafari A., Kamarehie B., Ghaderpoury A., Amini M.M. (2018). Ethylenediamine-functionalized cubic ZIF-8 for arsenic adsorption from aqueous solution: modeling, isotherms, kinetics and thermodynamics. J. Mol. Liq..

[2] Shams M., Dehghani M.H., Nabizadeh R., Mesdaghinia A., Alimohammadi M., Najafpoor A.A. (2016). Adsorption of phosphorus from aqueous solution by cubic zeolitic imidazolate framework-8: modeling, mechanical agitation versus sonication. J. Mol. Liq..

[3] Mohammadi A.A., Alinejad A., Kamarehie B., Javan S., Ghaderpoury A., Ahmadpour M., Ghaderpoori M. (2017). Metal organic framework Uio-66 for adsorption of methylene blue dye from aqueous solutions. Int. J. Environ. Sci. Technol..

[4] Massoudinejad M., Ghaderpoori M., Shahsavani A., Amini M.M. (2016). Adsorption of fluoride over a metal organic framework Uio-66 functionalized with amine groups and optimization with response surface methodology. J. Mol. Liq..

[5] Ghaderpoori M., Paydar M., Zarei A., Alidadi H., Najafpoor A.A., Gohary A.H., Shams M. (2018). Health risk assessment of fluoride in water distribution network of Mashhad, Iran. Hum. Ecol. Risk Assess.: Int. J..

[6] Rezaei H., Jafari A., Kamarehie B., Fakhri Y., Ghaderpoury A., Karami M.A., Ghaderpoori M., Shams M., Bidarpoor F., Salimi M. (2018). Health-risk assessment related to the fluoride, nitrate, and nitrite in the drinking water in the Sanandaj, Kurdistan County, Iran. Hum. Ecol. Risk Assess.: Int. J..

[7] Eaton A.D., Clesceri L.S., Rice E.W. (2012). Standard Methods for the Examination of Water and Wastewater.

[8] Ghaderpoori M., Khaniki G.R.J., Dehghani M., Shams M., Zarei A. (2009). Determination of fluoride in bottled water sold in Tehran market, Iran. Am.-Eurasian J. Agric. Environ. Sci..

[9] Saleh H.N., Dehghani M.H., Nabizadeh R., Mahvi A.H., Hossein F., Ghaderpoori M., Yousefi M., Mohammadi A. (2018). Data on the acid black 1 dye adsorbtion from aqueous solutions by low-cost adsorbent-Cerastoderma lamarcki shell collected from the northern coast of Caspian Sea. Data Brief.

[10] Ghaderpoori M., Jafari A., Ghaderpoury A. (2018). Heavy metals analysis and quality assessment in drinking water–Khorramabad city, Iran. Data Brief.

[11] Yazdanbakhsh A., Hashempour Y., Ghaderpouri M. (2018). Performance of granular activated carbon/nanoscale zero-valent iron for removal of humic substances from aqueous solution based on Experimental Design and Response Surface Modeling. Glob. NEST J..

[12] Habibi N., Rouhi P., Ramavandi B. (2017). Synthesis of adsorbent from Tamarix hispida and modified by lanthanum metal for fluoride ions removal from wastewater: adsorbent characteristics and real wastewater treatment data. Data Brief.

[13] Ravanipour M., Kafaei R., Keshtkar M., Tajalli S., Mirzaei N., Ramavandi B. (2017). Fluoride ion adsorption onto palm stone: optimization through response surface methodology, isotherm, and adsorbent characteristics data. Data Brief.

[14] Papari F., Najafabadi P.R., Ramavandi B. (2017). Fluoride ion removal from aqueous solution, groundwater, and seawater by granular and powdered Conocarpus erectus biochar. Desal Water Treat..

[15] Ramavandi B., Ahmadi M., Faradmal J., Maleki S., Asgari G. (2014). Optimization of fluoride adsorption from aqueous solution by marble powder using Taguchi model. J. Mazandaran Univ. Med. Sci..

[16] Papari F., Sahebi S., Kouhgardi E., Behresi R., Hashemi S., Asgari G., Jorfi S., Ramavandi B. (2017). Cyanide adsorption from aqueous solution using mesoporous zeolite modified by cetyltrimethylammonium bromide surfactant. Desalination Water Treat..

[17] Mohammadi A.A., Yousefi M., Yaseri M., Jalilzadeh M., Mahvi A.H. (2017). Skeletal fluorosis in relation to drinking water in rural areas of West Azerbaijan, Iran. Sci. Rep..

[18] Moghaddam V.K., Yousefi M., Khosravi A., Yaseri M., Mahvi A.H., Hadei M., Mohammadi A.A., Robati Z., Mokammel A. (2018). High concentration of fluoride can be increased risk of abortion. Biol. Trace Elem. Res..

[19] Asgari G., Ramavandi B., Rasuli L., Ahmadi M. (2013). Cr (VI) adsorption from aqueous solution using a surfactant-modified Iranian zeolite: characterization, optimization, and kinetic approach. Desalination Water Treat..

[20] Dehghani M.H., Zarei A., Mesdaghinia A., Nabizadeh R., Alimohammadi M., Afsharnia M. (2017). Response surface modeling, isotherm, thermodynamic and optimization study of arsenic (V) removal from aqueous solutions using modified bentonite-chitosan (MBC). Korean J. Chem. Eng..

[21] Dehghani M.H., Zarei A., Mesdaghinia A., Nabizadeh R., Alimohammadi M., Afsharnia M. (2017). Adsorption of Cr(VI) ions from aqueous systems using thermally sodium organo-bentonite biopolymer composite (TSOBC): response surface methodology, isotherm, kinetic and thermodynamic studies. Desalination Water Treat..

[22] Khosravi R., Zarei A., Heidari M., Ahmadfazeli A., Vosughi M., Fazlzadeh M. (2018). Application of ZnO and TiO_2_ nanoparticles coated onto montmorillonite in the presence of H_2_O_2_ for efficient removal of cephalexin from aqueous solutions. Korean J. Chem. Eng..

[23] Ghasemi S.M., Mohseni-Bandpei A., Ghaderpoori M., Fakhri Y., Keramati H., Taghavi M., Moradi B., Karimyan K. (2017). Application of modified maize hull for removal of cu(II)ions from aqueous solutions. Environ. Prot. Eng..

